# Micronutrient-Associated Single Nucleotide Polymorphism and Mental Health: A Mendelian Randomization Study

**DOI:** 10.3390/nu16132042

**Published:** 2024-06-27

**Authors:** Jingni Hui, Na Zhang, Meijuan Kang, Yifan Gou, Chen Liu, Ruixue Zhou, Ye Liu, Bingyi Wang, Panxing Shi, Shiqiang Cheng, Xuena Yang, Chuyu Pan, Feng Zhang

**Affiliations:** Key Laboratory of Trace Elements and Endemic Diseases of National Health and Family Planning Commission, School of Public Health, Health Science Center, Xi’an Jiaotong University, Xi’an 710061, China; xxjn357@163.com (J.H.); zhangna918116@163.com (N.Z.); kmj123456@stu.xjtu.edu.cn (M.K.); yfgou@stu.xjtu.edu.cn (Y.G.); liuchenlucia@163.com (C.L.); xjtuzrx@stu.xjtu.edu.cn (R.Z.); 3122115065@stu.xjtu.edu.cn (Y.L.); bingyiwang@stu.xjtu.edu.cn (B.W.); axingxing0813@163.com (P.S.); chengsq0701@stu.xjtu.edu.cn (S.C.); smile940323@stu.xjtu.edu.cn (X.Y.); panchuyu_dsa@163.com (C.P.)

**Keywords:** micronutrients, mental health, mendelian randomization, linkage disequilibrium score (LDSC) regression, causal relationship

## Abstract

Purpose: Previous studies have demonstrated the link between micronutrients and mental health. However, it remains uncertain whether this connection is causal. We aim to investigate the potential causal effects of micronutrients on mental health based on linkage disequilibrium score (LDSC) regression and Mendelian randomization (MR) analysis. Methods: Utilizing publicly available genome-wide association study (GWAS) summary datasets, we performed LDSC and MR analysis to identify candidate micronutrients with potential causal effects on mental health. Single nucleotide polymorphisms (SNPs) significantly linked with candidate micronutrients with a genome-wide significance level (*p* < 5 × 10^−8^) were selected as instrumental variables (IVs). To estimate the causal effect of candidate micronutrients on mental health, we employed inverse variance weighted (IVW) regression. Additionally, two sensitivity analyses, MR-Egger and weighted median, were performed to validate our results. Results: We found evidence supporting significant causal associations between micronutrients and mental health. LDSC detected several candidate micronutrients, including serum iron (genetic correlation = −0.134, *p* = 0.032) and vitamin C (genetic correlation = −0.335, *p* < 0.001) for attention-deficit/hyperactivity disorder (ADHD), iron-binding capacity (genetic correlation = 0.210, *p* = 0.037) for Alzheimer’s disease (AD), and vitamin B12 (genetic correlation = −0.178, *p* = 0.044) for major depressive disorder (MDD). Further MR analysis suggested a potential causal relationship between vitamin B12 and MDD (b = −0.139, *p* = 0.009). There was no significant heterogeneity or pleiotropy, indicating the validity of the findings. Conclusion: In this study, we identified underlying causal relationships between micronutrients and mental health. Notably, more research is necessary to clarify the underlying biological mechanisms by which micronutrients affect mental health.

## 1. Introduction

The Global Burden of Disease Study (GBD) in 2016 demonstrated that neurological disorders were the primary contributors to disability-adjusted life years (DALYs) and occupied the second position in causing deaths [1]. GBD 2019 illustrated that psychiatric disorders continued to be the predominant factors of burden worldwide [2]. These disorders are linked with substantial morbidity and elevated mortality rates attributed to suicide and poor physical well-being [3], creating a heavy burden on society. While previous studies have demonstrated that all common mental health disorders have a substantial heritability [4,5], the pathogenic mechanisms of these disorders remain elusive. Despite their high prevalence, few risk factors have been established, making it critical to identify modifiable factors that can be targeted for prevention.

The etiology of mental health is complex, and the pathogenesis is unclear. Vitamins and minerals, also known as micronutrients, play a crucial role in supporting brain health and maintaining optimal cognitive function within the central nervous system [6]. Notably, micronutrient deficiencies are common public health concerns in low-income regions, particularly, iron, vitamin B12, and vitamin C were reported. Iron deficiency is one of the most common nutritional deficiencies worldwide, affecting an estimated one-third of the global population [7]. It is estimated that 1.5% of the general population may have suboptimal levels of vitamin B12 [8], and more than 7% of the United States population is deficient in vitamin C [9]. Observational studies have shown the relationship between micronutrients and mental health disorders. For instance, children with iron deficiency anemia are more likely to experience developmental delays and behavioral disturbances [10,11]. Maintaining higher serum B12 levels in older adults protects against Alzheimer’s disease (AD) [12]. Nutrient supplements have the potential to treat and alleviate mental health disorders [13]. However, causal evidence on the link between micronutrients and mental health disorders is still lacking.

Genome-wide association studies (GWASs) are a powerful tool in understanding the genetic underpinnings of micronutrient metabolism and deficiency. GWASs have pinpointed noteworthy single nucleotide polymorphisms (SNPs) linked to micronutrient insufficiency on a genome-wide scale [14]. Linkage disequilibrium score (LDSC) regression serves as a potent method for gauging genetic correlations amid multiple human complex traits utilizing GWAS summary data [15]. Mendelian randomization (MR) is an epidemiological approach used to assess the causal relationship between exposure factors and outcomes [16]. MR uses genetic variation, specifically, single nucleotide polymorphisms (SNPs), as effective instrumental variables (IVs) to avoid the influence of confounding factors in observational studies [16]. The integration of genetic insights into public health and clinical practices is paving the way for personalized and precision nutrition interventions.

In this study, we combined LDSC regression and MR analysis to evaluate the causal relationship between candidate micronutrients and multiple mental health disorders, providing new insights for risk management and preventive interventions for mental disorders.

## 2. Methods

### 2.1. Study Design

**Figure 1:**
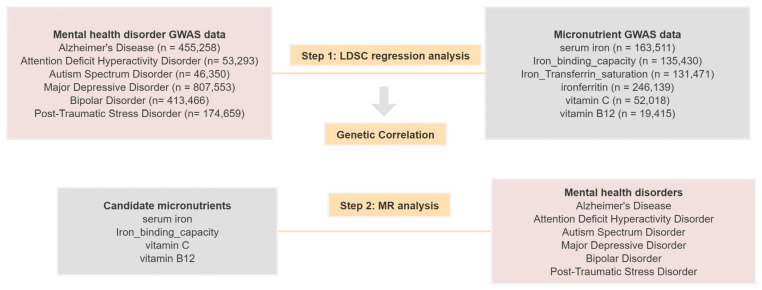
Study design of this study.

**Figure 2:**
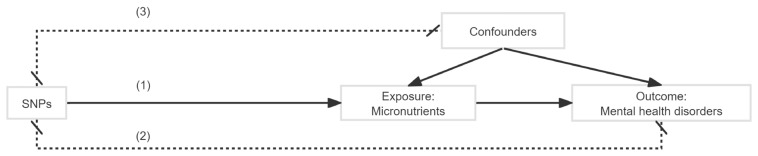
The basic principles of Mendelian randomization. Mendelian randomization can be used to evaluate the relationship between micronutrients and disorders, provided that the following three key assumptions are met adequately: (1) the SNPs are robustly associated with micronutrients; (2) the SNPs are not associated with confounders; (3) the SNPs have no association with the disorders except through the micronutrients.

Figure 1 illustrates the comprehensive design of this study, and Figure 2 depicts the fundamental principles of MR analysis. Briefly, SNPs should satisfy three basic conditions [17]. First, SNPs must be significantly associated with micronutrients. Second, SNPs should not have a direct effect on mental health outcomes but should influence the outcome solely through the exposure variable. Third, SNPs should be independent of any confounders. Our study first performed LDSC regression to explore the genetic correlation between mental health disorders and micronutrients. To validate the causality between candidate micronutrients and mental health, we further conducted an MR analysis.

### 2.2. GWAS Summary Datasets for Micronutrients

GWAS summary datasets for micronutrients were derived from previous studies, which are available on websites (https://www.ebi.ac.uk/gwas/, accessed on 29 March 2023). Micronutrients of interest were included in this study based on a comprehensive review of the existing literature and the availability of robust GWAS data. Specific micronutrients of interest were significantly associated with the risk of mental health disorders identified in previous studies, including iron [18], vitamin A [19], vitamin B6 [19], folic acid [19], vitamin B12 [19], vitamin C [20], and vitamin D [21]. The GWAS summary datasets for vitamin A, vitamin B6, and folic acid were excluded because there were few significant SNPs closely correlated with them at a level of genome-wide significance (*p* < 5 × 10^−8^). While the SNPs of vitamin D were removed because of linkage disequilibrium (LD) with other variants or absence from the LD reference panel. We ultimately selected iron, vitamin C, and vitamin B12 as variable instruments for the LDSC regression and MR analyses. More detailed information is available in the original articles [18,19,20,21].

### 2.3. GWAS Summary Data for Mental Health

The mental health disorders included in our study were selected based on their high public health burden, the availability of genetic data, and the known or hypothesized association with micronutrient levels. Six neuropsychiatric traits were enrolled from the largest recent GWASs, including Alzheimer’s disease (AD) [22], attention-deficit/hyperactivity disorder (ADHD) [23], autism spectrum disorder (ASD) [24], major depressive disorder (MDD) [25], bipolar disorder (BIP) [26], and post-traumatic stress disorder (PTSD) [27]. The GWAS summary data for these disorders were acquired from the Psychiatric Genomics Consortium (PGC) website (https://www.med.unc.edu/pgc, accessed on 6 April 2023). The GWAS data for six mental health disorders is as follows: 455,258 AD individuals (71,880 cases and 383,378 controls); 53,293 ADHD individuals (19,099 cases and 34,194 controls); 46,350 ASD individuals (18,381 cases and 27,969 controls); 807,553 MDD individuals (246,363 cases and 561,190 controls); 413,466 BIP individuals (41,917 cases and 371,549 controls); and 174,659 PTSD individuals (23,212 PTSD cases and 151,447 controls). All subjects were of European descent. Details about genotyping, imputation, quality control, and genetic association analysis were described in the primary studies [22,23,24,25,26,27].

### 2.4. LDSC Regression Analysis

To calculate the genetic correlation of micronutrients with six mental health disorders, we performed LDSC regression analysis with the summary statistics. Firstly, we reformatted GWAS summary statistics utilizing munge_sumstats.py (https://github.com/bulik/ldsc/blob/master/munge_sumstats.py, accessed on 5 May 2023). Variants with non-SNPs as well as ambiguous and repeated SNPs were eliminated. To mitigate bias due to differences in imputation quality, we selected SNPs with an imputation quality score > 0.9 and minor allele frequency (MAF) > 0.01. Subsequently, we used the standard approach of the 1000 Genomes Project as a reference panel of linkage disequilibrium (LD) for LD score estimation (clump_r^2^ = 0.01, clump_kb = 5000, *p* = 1 × 10^−5^). Finally, LDSC was carried out to evaluate the genetic correlation between micronutrients and mental health disorders (https://github.com/bulik/ldsc, accessed on 5 May 2023) (LDSCore v1.0.1).

### 2.5. Genetic Instruments Selection

MR analysis utilized genetic variants linked to exposure as instrumental variables (IVs). This current study identified candidate micronutrients correlated with mental health through LDSC regression analysis. Subsequently, SNPs associated with candidate micronutrients of interest in GWASs but not directly with confounders were employed as genetic instruments. To ensure that the SNPs selected as IVs were strongly linked to micronutrients, independent SNPs related to micronutrients were involved in this study with a genome-wide significant level (*p* < 5 × 10^−8^).

### 2.6. MR Analysis

In this study, we employed MR analysis to evaluate the causation among multiple candidate micronutrients and mental health disorders. The inverse variance weighted (IVW) [28] was utilized to determine the causal effects of candidate micronutrients on mental health disorders. To improve the credibility and robustness of our findings, we further considered MR-Egger [29] and weighted median [30] as sensitivity analyses. Q-tests were performed to assess potential heterogeneity with *p* > 0.05 indicating no heterogeneity among SNPs. The MR-Egger intercept was examined to detect any potential horizontal pleiotropy among the instrument variables. Weighted median yields consistent estimates when at least half of the information originates from valid instrumental variables. Additionally, MR-PRESSO [31] was applied to estimate and rectify pleiotropy by excluding outliers from the IVW model.

All statistical analyses were conducted using R (version 4.0.2). The IVW, weighted median, and MR-Egger regression methods were implemented with the “TwoSampleMR” package (version 4.0.2), while the MR-PRESSO test was executed with the “MRPRESSO” package.

## 3. Results

### 3.1. Genetic Correlation between Micronutrients and Mental Health

The GWAS data source of micronutrients are summarized in Table 1, and the neuropsychiatric traits are summarized in Table 2. LDSC detected several candidate genetic correlations between micronutrients and mental health, such as serum iron (genetic correlation = −0.134, *p* = 0.032) and vitamin C (genetic correlation = −0.335, *p* < 0.001) for ADHD, iron-binding capacity (genetic correlation = 0.210, *p* = 0.037) for AD, and vitamin C (genetic correlation = −0.165, *p* < 0.001) and vitamin B12 (genetic correlation = −0.178, *p* = 0.044) for MDD. Supplementary Table S1 displays the results of LDSC regression analysis.

### 3.2. MR Estimates of the Causality between Vitamin B12 and Mental Health

Both LDSC regression analysis (genetic correlation = −0.178, *p* = 0.044) and the IVW method showed vitamin B12 (b = −0.139, *p* = 0.009, Table 3) was negatively associated with MDD. In addition, weighted median estimates suggested that the vitamin B12 level was causally associated with MDD (*P*_weighted median_ = 0.001, Table 3). The IVW results also showed a positive correlation between the genetically predicted vitamin B12 and ASD (b = 0.205, *p* = 0.019, Table 3). There was no evidence linking vitamin B12 with other mental health disorders (Supplementary Table S4).

For significant results, Cochran’s IVW-Q test indicated no significant heterogeneity in the effect of vitamin B12 on ASD (*p* = 0.608) and MDD (*p* = 0.176). The MR-Egger analysis confirmed no notable horizontal pleiotropy in our study (*P*_ASD_ = 0.965, *P*_MDD_ = 0.738). Additionally, the MR-PRESSO global test found no significant outliers (global test *P*_ASD_ = 0.248, *P*_MDD_ = 0.128, Table 3).

### 3.3. MR Estimates of the Causality between Iron and Mental Health

The IVW results showed that the iron-binding capacity level was positively correlated with ASD (b = 0.088, *p* = 0.027, Table 3). Although IVW estimates did not support the causal associations between the iron-binding capacity level and MDD (*P*_IVW_ = 0.481) and PTSD (*P*_IVW_ = 0.061), weighted median estimates suggested that the iron-binding capacity level was causally associated with MDD (*P*_weighted median_ = 0.010) and PTSD (*P*_weighted median_ = 0.028, Supplementary Table S2). There was no evidence that iron-related indicators were associated with other mental health disorders (Supplementary Table S2).

For significant results, Cochran’s IVW Q-test indicated no significant heterogeneity in the iron-related variables (*P*_ASD_ = 0.272). The MR-Egger analysis confirmed no notable horizontal pleiotropy in our study (*P*_ASD_ = 0.836). Additionally, the MR-PRESSO global test found no significant outliers (global test *P*_ASD_ = 0.125, Table 3).

### 3.4. MR Estimates of the Causality between Vitamin C and Mental Health

The IVW results suggested a negative correlation between genetically predicted vitamin C and AD (b = −0.036, *p* = 0.032, Table 3). Weighted median estimates suggested that vitamin C was causally linked to AD (*P*_weighted median_ = 0.023, Table 3). There was no evidence that genetically predicted vitamin C was related to other mental health disorders (Supplementary Table S3).

Cochran’s IVW Q-test displayed significant heterogeneity in the effect of vitamin C on AD (*p* = 0.039). The MR-Egger analysis confirmed no notable horizontal pleiotropy in our study (*p* = 0.703). Additionally, the MR-PRESSO global test detected significant outliers (global test *p* = 0.015, Table 3).

## 4. Discussion

Using publicly available GWAS summary datasets, we identified independent SNPs associated with each mental health disorder by LD analysis. In addition, we used MR analysis to investigate the causal effects of micronutrients on six mental health disorders, providing new views into the causal links among them.

Vitamin B12 is a necessary micronutrient to maintain proper neurological function, as it is involved in homocysteine regeneration, methionine synthesis, methylation process, and the synthesis of serotonin (5-HT). Low vitamin B12 levels can interfere with homocysteine regeneration to methionine and methylmalonyl-CoA (MMA) isomerization to succinyl-CoA, resulting in increased circulating levels of homocysteine and MMA [32]. The increase in homocysteine can also raise the level of reactive oxygen and subsequently contribute to neuronal apoptosis, thus inducing depressive symptoms [33]. Increasing evidence links vitamin B12 deficiency to a higher risk of depression [34]. Maryam et al. found that a healthy diet, which raises serum vitamin B12 levels, was correlated with a decreased risk of depression [35]. Our study found that an elevated circulating vitamin B12 level was linked to a lower risk of MDD. One study suggested a “U”-shaped correlation between the frequency of maternal multivitamin supplementation and ASD risk, with both very high and very low maternal plasma vitamin B12 levels being related to ASD. Vitamin B12 is crucial for DNA methylation, cellular growth, and differentiation [36]. The connection between vitamin B12 deficiency and ASD could be attributed to DNA hypomethylation, which impacts CNS development [37]. Our study detected a correlation between elevated vitamin B12 levels and an increased risk of ASD.

Substantial evidence highlights the crucial importance of iron in the cognitive, behavioral, and physical development of children [38]. Dysregulation in iron homeostasis has been linked to several mental health disorders such as depression [39], anxiety [40], and schizophrenia [41]. Elevated iron deposition in the brain has been observed in older adult humans and persons with Alzheimer’s disease (AD) and has been associated with lower cognitive performance [42]. In this study, we observed that the genetically predicated iron-binding capacity level was correlated with a heightened risk of ASD. Iron is intricately connected to dopaminergic neurotransmission, as well as dopamine synthesis [43]. The accumulation of iron might trigger neurotoxicity and synaptic toxicity via oxidative stress and ferroptosis, leading to overactive dopamine neurotransmission and ultimately contributing to psychiatric disorders [44,45,46].

Increasing evidence indicates a crucial role of vitamin C in AD pathogenesis. Vitamin C is a powerful antioxidant, supporting neurodevelopment, regulating neurotransmitters, protecting neurons from oxidative stress-induced damage, and maintaining the normal function of the nervous system. In addition to reducing oxidative stress, vitamin C is crucial in inhibiting pro-inflammatory genes, neuroinflammation, and Aβ fibrillary genesis [47,48]. The correlation between plasma vitamin C levels and AD was found in an observational study [49]. A meta-analysis demonstrated that the plasma level of Vitamin C was significantly lower in patients with AD [50]. One MR study suggested an inverse correlation linking vitamin C levels with AD risk [51], consistent with our MR results.

This study systematically assessed the causal relationship between various micronutrients and multiple mental health disorders using Mendelian randomization methods based on available summary data. Mendelian randomization leverages SNPs, associated with the micronutrients of interest but not directly with confounders, as instrumental variables, ensuring the SNPs are valid instruments and allowing for more robust causal inferences compared with observational studies, which are often confounded by external factors. Moreover, MR findings might diverge from observational studies in minimizing the influence of confounding factors and reverse causation.

Nevertheless, some constraints were inevitable in this investigation. Firstly, the GWAS summary data limited our ability to evaluate the influence of population stratification. Moreover, because of the insufficient demographic information, further subgroup analyses were unfeasible. Secondly, the presence of weak instruments could potentially amplify the correlation between micronutrients and disorders. Furthermore, the GWASs of some micronutrients (vitamin B6, vitamin A, and serum folate) could not be used to perform MR analysis because of inadequate significant SNPs, underscoring the necessity for larger-scale GWASs to investigate the causal link between micronutrients with mental health disorders comprehensively. Thirdly, horizontal pleiotropy, a common challenge in MR, is hard to avoid. To mitigate the horizontal pleiotropy, we employed MR-Egger and MR-PRESSO. Moreover, hormonal changes might play a role in mediating the relationship between micronutrients and mental health disorders. There is a potential need for future research in this area. Finally, mental health disorders are influenced by multiple factors, involving genetic and environmental elements, with micronutrients playing only part of the role, significantly affected by genetics.

Conclusively, we identified underlying causal relationships between micronutrients and mental health disorders. In particular, both LDSC and MR methods found a negative relationship between vitamin B12 and MDD. Moreover, MR analysis identified the causal link between vitamin C and AD, as well as iron status and ASD. Further investigation is required to ascertain our discoveries and to obtain a deeper understanding of the underlying mechanisms.

## Figures and Tables

**Table 1:** Dataset of the micronutrient information.

Exposure	GWAS Data Source	Sample Size
iron		
ironferritin	Bell S et al., 2021 [18]	246,139
iron-binding capacity	Bell S et al., 2021 [18]	135,430
serum iron	Bell S et al., 2021 [18]	163,511
iron transferrin saturation	Bell S et al., 2021 [18]	131,471
vitamin A	Dennis JK et al., 2021 [19]	2007
vitamin B6	Dennis JK et al., 2021 [19]	1758
folic acid	Dennis JK et al., 2021 [19]	4409
vitamin B12	Dennis JK et al., 2021 [19]	19,415
vitamin C	Zheng JS et al., 2021 [20]	52,018
vitamin D	Manousaki D et al., 2020 [21]	443,734

**Table 2:** Dataset of neuropsychiatric disorder information.

Disease		Sample Size	
	Total	Cases	Controls
AD	455,258	71,880	383,378
ADHD	53,293	19,099	34,194
ASD	46,350	18,381	27,969
MDD	807,553	246,363	561,190
BIP	413,466	41,917	371,549
PTSD	174,659	23,212	151,447

Abbreviations: AD, Alzheimer’s disease; ADHD, attention-deficit/hyperactivity disorder; ASD, autism spectrum disorder; MDD, major depressive disorder; BIP, bipolar disorder; PTSD, post-traumatic stress disorder.

**Table 3:** MR estimates of the causal effects between micronutrients and disorders.

Exposure	Outcome	Method	nSNP	b	SE	*p*-Value	Heterogeneity	Pleiotropy	MR-PRESSO
TIBC	ASD	MR-Egger	51	0.099	0.066	0.138	0.242	0.836	0.125
		Weighted median	51	0.083	0.057	0.144			
		IVW	51	0.088	0.040	0.027	0.272		
Vitamin C	AD	MR-Egger	10	−0.027	0.028	0.372	0.027	0.703	0.015
		Weighted median	10	−0.037	0.016	0.023			
		IVW	10	−0.036	0.017	0.032	0.039		
Vitamin B12	ASD	MR-Egger	5	0.183	0.461	0.718	0.439	0.965	0.248
		Weighted median	5	0.182	0.114	0.111			
		IVW	5	0.205	0.087	0.019	0.608		
Vitamin B12	MDD	MR-Egger	3	−0.260	0.285	0.530	0.087	0.738	0.128
		Weighted median	3	−0.178	0.055	0.001			
		IVW	3	−0.139	0.054	0.009	0.176		

Note: Abbreviations: TIBC, iron-binding capacity.

## Data Availability

The datasets used and/or analyzed during the current study are available from the corresponding author upon reasonable request.

## Supplementary Materials

The following supporting information can be downloaded at https://www.mdpi.com/article/10.3390/nu16132042/s1, Table S1: Candidate genetic correlations between micronutrients and disorders. Table S2: MR estimates of the causality between iron-related traits and disorders. Table S3: MR estimates of the causality between vitamin C and disorders. Table S4: MR estimates of the causality between vitamin B12 and disorders.
