# Supplementary material for: Micronutrient-Associated Single Nucleotide Polymorphism and Mental Health: A Mendelian Randomization Study
Source: Nutrients. 2024 Jun 27;16(13):2042. doi: 10.3390/nu16132042 (PMC11243241; doi:10.3390/nu16132042)
Supplement: Supplementary file 1 [file nutrients-16-02042-s001.zip › nutrients-3032553-supplementary.pdf]

Supplement Table S1. candidate genetic correlations between micronutrients and mental health disorders

	**Disorder**	**Genetic correlation**	***P***
iron serum	AD	-0.083	0.401
	ADHD	-0.134	0.032
	ASD	-0.109	0.127
	MDD	-0.035	0.408
	BIP	0.080	0.183
	PTSD	-0.157	0.139
TIBC	AD	0.210	0.037
	ADHD	0.023	0.661
	ASD	0.010	0.874
	MDD	0.088	0.017
	BIP	-0.023	0.628
	PTSD	0.235	0.009
TSAT	AD	-0.181	0.083
	ADHD	-0.084	0.169
	ASD	-0.073	0.371
	MDD	-0.062	0.126
	BIP	0.093	0.120
	PTSD	-0.180	0.082
ironferritin	AD	0.053	0.455
	ADHD	0.014	0.728
	ASD	-0.033	0.517
	MDD	-0.036	0.184
	BIP	-0.033	0.312
	PTSD	-0.117	0.111
vitamin C	AD	-0.067	0.479
	ADHD	-0.335	<0.001
	ASD	-0.069	0.382
	MDD	-0.165	<0.001
	BIP	0.211	<0.001
	PTSD	-0.153	0.141
vitamin B12	AD	-0.446	0.064
	ADHD	-0.054	0.640
	ASD	0.142	0.293
	MDD	-0.178	0.044
	BIP	-0.034	0.747
	PTSD	-0.355	0.132

Note: Abbreviations:AD, Alzheimer’s disease; ADHD, attention-deficit/hyperactivity disorder; ASD, autism spectrum disorder; BIP, bipolar disorder; MDD, major depression disorder; PTSD, post-traumatic stress disorder; TIBC, Iron binding capacity; TSAT,Iron_Transferrin_saturation.

Supplement Table S2. MR estimates of the causality between iron and mental health disorders

Exposure	Outcome	Method	nSNP	b	SE	P value	OR	95% LCI	95% UCI	Heterogeity	Horizontal pleiotropy	MR-PRESSO global test
ironferritin	AD	MR Egger	70	-0.006	0.021	0.791	0.994	0.954	1.037	0.013	0.989	0.013
		Weighted median	70	-0.012	0.014	0.401	0.988	0.960	1.016			
		IVW	70	-0.006	0.011	0.579	0.994	0.974	1.015	0.016		
ironferritin	ADHD	MR Egger	63	-0.034	0.132	0.798	0.967	0.747	1.251	0.056	0.562	0.042
		Weighted median	63	0.044	0.090	0.624	1.045	0.876	1.247			
		IVW	63	0.032	0.066	0.623	1.033	0.908	1.176	0.063		
ironferritin	ASD	MR Egger	69	0.014	0.114	0.900	1.014	0.811	1.269	0.555	0.965	0.620
		Weighted median	69	0.092	0.085	0.279	1.097	0.928	1.296			
		IVW	69	0.019	0.057	0.744	1.019	0.910	1.140	0.590		
ironferritin	MDD	MR Egger	52	-0.137	0.112	0.228	0.872	0.700	1.087	<0.001	0.278	<0.001
		Weighted median	52	0.046	0.043	0.285	1.047	0.963	1.138			
		IVW	52	-0.029	0.054	0.591	0.971	0.873	1.080	<0.001		
ironferritin	BIP	MR Egger	67	0.047	0.126	0.710	1.048	0.818	1.343	0.032	0.622	0.061
		Weighted median	67	0.118	0.092	0.202	1.125	0.939	1.348			
		IVW	67	-0.006	0.065	0.922	0.994	0.875	1.129	0.036		
ironferritin	PTSD	MR Egger	70	0.059	0.096	0.544	1.061	0.878	1.281	0.353	0.255	0.372
		Weighted median	70	0.011	0.076	0.886	1.011	0.872	1.173			
		IVW	70	-0.035	0.052	0.502	0.966	0.873	1.069	0.342		
ironserum	AD	MR Egger	41	-0.003	0.022	0.906	0.997	0.955	1.041	<0.001	0.400	0.002
		Weighted median	41	-0.021	0.013	0.116	0.979	0.954	1.005			
		IVW	41	-0.018	0.013	0.171	0.982	0.958	1.008	<0.001		
ironserum	ADHD	MR Egger	32	0.146	0.095	0.135	1.157	0.961	1.393	0.466	0.200	0.472
		Weighted median	32	0.063	0.075	0.405	1.065	0.919	1.234			
		IVW	32	0.047	0.058	0.421	1.048	0.935	1.174	0.431		
ironserum	ASD	MR Egger	37	0.009	0.112	0.937	1.009	0.810	1.256	0.076	0.620	0.111
		Weighted median	37	0.112	0.090	0.214	1.119	0.937	1.336			
		IVW	37	0.054	0.065	0.408	1.055	0.929	1.199	0.088		
ironserum	MDD	MR Egger	29	-0.040	0.101	0.698	0.961	0.788	1.172	<0.001	0.400	<0.001
		Weighted median	29	-0.028	0.031	0.369	0.973	0.916	1.033			
		IVW	29	0.028	0.063	0.654	1.028	0.910	1.163	<0.001		
ironserum	BIP	MR Egger	37	-0.099	0.092	0.288	0.906	0.756	1.084	0.378	0.172	0.235
		Weighted median	37	-0.014	0.083	0.869	0.986	0.839	1.160			
		IVW	37	0.002	0.057	0.969	1.002	0.896	1.121	0.335		
ironserum	PTSD	MR Egger	39	0.061	0.097	0.537	1.063	0.878	1.286	0.301	0.379	0.395
		Weighted median	39	-0.002	0.075	0.983	0.998	0.862	1.156			
		IVW	39	-0.010	0.056	0.853	0.990	0.887	1.104	0.307		
TSAT	AD	MR Egger	49	-0.010	0.016	0.542	0.990	0.959	1.022	<0.001	0.671	<0.001
		Weighted median	49	-0.005	0.009	0.573	0.995	0.977	1.013			
		IVW	49	-0.005	0.010	0.650	0.996	0.976	1.015	<0.001		
TSAT	ADHD	MR Egger	36	0.065	0.079	0.413	1.067	0.915	1.245	0.206	0.761	0.247

		Weighted median	36	0.069	0.058	0.239	1.071	0.955	1.201			
		IVW	36	0.046	0.047	0.329	1.047	0.955	1.148	0.237		
TSAT	ASD	MR Egger	43	-0.001	0.077	0.987	0.999	0.858	1.163	0.228	0.885	0.139
		Weighted median	43	-0.023	0.063	0.720	0.978	0.864	1.106			
		IVW	43	-0.010	0.046	0.821	0.990	0.905	1.082	0.261		
TSAT	MDD	MR Egger	35	0.013	0.074	0.857	1.013	0.877	1.171	<0.001	0.961	<0.001
		Weighted median	35	0.000	0.022	0.989	1.000	0.957	1.045			
		IVW	35	0.016	0.045	0.718	1.016	0.931	1.110	<0.001		
TSAT	BIP	MR Egger	40	-0.144	0.072	0.052	0.866	0.753	0.997	0.349	0.193	0.158
		Weighted median	40	-0.108	0.058	0.063	0.898	0.801	1.006			
		IVW	40	-0.068	0.043	0.119	0.934	0.858	1.018	0.316		
TSAT	PTSD	MR Egger	47	-0.053	0.070	0.449	0.948	0.827	1.087	0.300	0.741	0.208
		Weighted median	47	-0.092	0.060	0.122	0.912	0.811	1.025			
		IVW	47	-0.035	0.041	0.400	0.966	0.891	1.047	0.333		
TIBC	AD	MR Egger	49	0.002	0.013	0.862	1.002	0.978	1.027	0.001	0.935	<0.001
		Weighted median	49	-0.002	0.009	0.855	0.998	0.980	1.017			
		IVW	49	0.003	0.008	0.706	1.003	0.987	1.019	0.001		
TIBC	ADHD	MR Egger	42	0.067	0.065	0.315	1.069	0.940	1.215	0.244	0.565	0.456
		Weighted median	42	0.002	0.061	0.972	1.002	0.890	1.128			
		IVW	42	0.037	0.041	0.363	1.038	0.958	1.123	0.267		
TIBC	ASD	MR Egger	51	0.099	0.066	0.138	1.104	0.971	1.255	0.242	0.836	0.125
		Weighted median	51	0.083	0.057	0.144	1.086	0.972	1.214			
		IVW	51	0.088	0.040	0.027	1.092	1.010	1.181	0.272		
TIBC	MDD	MR Egger	37	0.093	0.072	0.207	1.098	0.952	1.265	<0.001	0.285	<0.001
		Weighted median	37	0.052	0.020	0.010	1.053	1.013	1.096			
		IVW	37	0.033	0.047	0.481	1.034	0.943	1.133	<0.001		
TIBC	BIP	MR Egger	45	0.051	0.082	0.534	1.053	0.897	1.236	0.001	0.745	0.002
		Weighted median	45	0.007	0.062	0.905	1.007	0.892	1.138			
		IVW	45	0.031	0.051	0.549	1.031	0.933	1.140	0.001		
TIBC	PTSD	MR Egger	52	0.115	0.056	0.045	1.122	1.005	1.252	0.641	0.258	0.420
		Weighted median	52	0.110	0.050	0.028	1.117	1.012	1.232			
		IVW	52	0.065	0.035	0.061	1.067	0.997	1.142	0.628		

Supplement Table S3. MR estimates of the causality between Vitamin C and mental health disorders

Exposure	Outcome	Method	nSNP	b	SE	P value	OR	95% LCI	95% UCI	Heterogeity	Horizontal pleiotropy	MR-PRESSO global test
Vitamin C	AD	MR Egger	10	-0.027	0.028	0.372	0.974	0.921	1.029	0.027	0.703	0.015
		Weighted median	10	-0.037	0.016	0.023	0.963	0.933	0.995			
		IVW	10	-0.036	0.017	0.032	0.965	0.934	0.997	0.039		
Vitamin C	ADHD	MR Egger	7	0.022	0.115	0.858	1.022	0.815	1.280	0.453	0.646	0.608
		Weighted median	7	0.019	0.093	0.838	1.019	0.850	1.222			
		IVW	7	0.063	0.077	0.411	1.065	0.916	1.239	0.551		
Vitamin C	ASD	MR Egger	10	0.039	0.116	0.747	1.039	0.828	1.305	0.424	0.546	0.587
		Weighted median	10	-0.029	0.099	0.767	0.971	0.801	1.178			
		IVW	10	-0.017	0.075	0.819	0.983	0.849	1.138	0.485		
Vitamin C	MDD	MR Egger	9	-0.009	0.080	0.912	0.991	0.847	1.160	<0.001	0.660	<0.001
		Weighted median	9	0.020	0.030	0.511	1.020	0.962	1.082			
		IVW	9	0.019	0.049	0.693	1.019	0.927	1.121	<0.001		
Vitamin C	BIP	MR Egger	10	-0.301	0.196	0.164	0.740	0.504	1.088	0.003	0.015	0.010
		Weighted median	10	-0.145	0.094	0.126	0.865	0.719	1.041			
		IVW	10	-0.050	0.137	0.716	0.951	0.727	1.245	<0.001		
Vitamin C	PTSD	MR Egger	10	-0.089	0.097	0.383	0.915	0.757	1.105	0.739	0.144	0.602
		Weighted median	10	-0.022	0.074	0.766	0.978	0.846	1.131			
		IVW	10	0.028	0.064	0.663	1.028	0.907	1.166	0.555		

Supplement Table S4. MR estimates of the causality between Vitamin B12 and mental health disorders

Exposure	Outcome	Method	nSNP	b	SE	P value	OR	95% LCI	95% UCI	Heterogeity	Horizontal pleiotropy	MR-PRESSO global test
Vitamin B12	AD	MR Egger	5	0.101	0.073	0.258	1.107	0.960	1.277	0.857	0.208	0.060
		Weighted median	5	-0.017	0.018	0.330	0.983	0.949	1.018			
		IVW	5	-0.013	0.014	0.368	0.987	0.960	1.015	0.504		
Vitamin B12	ADHD	MR Egger	5	0.898	0.694	0.286	2.456	0.630	9.572	0.070	0.318	0.299
		Weighted median	5	0.032	0.126	0.799	1.033	0.806	1.322			
		IVW	5	0.085	0.140	0.544	1.089	0.827	1.432	0.034		
Vitamin B12	ASD	MR Egger	5	0.183	0.461	0.718	1.201	0.487	2.962	0.439	0.965	0.248
		Weighted median	5	0.182	0.114	0.111	1.200	0.959	1.501			
		IVW	5	0.205	0.087	0.019	1.227	1.034	1.456	0.608		
Vitamin B12	MDD	MR Egger	3	-0.260	0.285	0.530	0.771	0.441	1.349	0.087	0.738	0.128
		Weighted median	3	-0.178	0.055	0.001	0.837	0.752	0.931			
		IVW	3	-0.139	0.054	0.009	0.870	0.783	0.966	0.176		
Vitamin B12	BIP	MR Egger	5	-0.482	0.701	0.541	0.618	0.156	2.440	0.072	0.684	0.007
		Weighted median	5	-0.144	0.117	0.217	0.865	0.688	1.089			
		IVW	5	-0.173	0.118	0.144	0.841	0.667	1.061	0.114		
Vitamin B12	PTSD	MR Egger	5	-0.303	0.461	0.558	0.738	0.299	1.824	0.918	0.620	0.953
		Weighted median	5	-0.069	0.096	0.471	0.933	0.774	1.126			
		IVW	5	-0.053	0.083	0.520	0.948	0.806	1.116	0.938		
